# Loss of PR55α promotes proliferation and metastasis by activating MAPK/AKT signaling in hepatocellular carcinoma

**DOI:** 10.1186/s12935-021-01796-0

**Published:** 2021-02-15

**Authors:** JiangSheng Zhao, GuoFeng Chen, Jingqi Li, Shiqi Liu, Quan Jin, ZhengWei Zhang, Fuzhen Qi, JianHuai Zhang, JianBo Xu

**Affiliations:** 1 Department of Hepatobiliary Surgery, The Affiliated Huaian NO.1 People’s Hospital of Nanjing Medical University, Huai’an, 223001 Jiangsu People’s Republic of China; 2 Department of Pathology, The Affiliated Huaian NO.1 People’s Hospital of Nanjing Medical University, Huai’an, 223001 Jiangsu People’s Republic of China

**Keywords:** Hepatocellular carcinoma, PR55α, Cell proliferation, Cancer metastasis, MAPK/AKT signaling pathway

## Abstract

**Background:**

PR55α plays important roles in oncogenesis and progression of numerous malignancies. However, its role in hepatocellular carcinoma (HCC) is unclear. This study aims to characterize the functions of PR55α in HCC.

**Methods:**

PR55α expressions in HCC tissues and paired healthy liver samples were evaluated using Western blot and tissue microarray immunohistochemistry. We knocked down the expression of PR55α in SMMC-7721 and LM3 cell lines via small interfering and lentivirus. In vitro cell counting, colony formation, migration and invasion assays were performed along with in vivo xenograft implantation and lung metastases experiments. The potential mechanisms involving target signal pathways were investigated by RNA-sequencing.

**Results:**

PR55α expression level was suppressed in HCC tissues in comparison to healthy liver samples. Decreased PR55α levels were correlated with poorer prognosis (P = 0.0059). Knockdown of PR55α significantly promoted cell proliferation and migration, induced repression of the cell cycle progression and apoptosis in vitro while accelerating in vivo HCC growth and metastasis. Mechanistic analysis indicated that PR55α silencing was involved with MAPK/AKT signal pathway activation and resulted in increased phosphorylation of both AKT and ERK1/2.

**Conclusions:**

This study identifies PR55α to be a candidate novel therapeutic target in the treatment of HCC.

## Introduction

Hepatocellular carcinoma (HCC) represents the seventh most common and the third most fatal malignant tumor [1]. Despite improvements in early diagnostic methods and advanced surgical and medical therapy, HCC carries an abysmally poor prognosis, with less than 10% of patients surviving more than 5 years from diagnosis [2]. It is therefore of utmost importance that improved clinical diagnostic and treatment methods are established.

Inactivation of tumor suppressor genes is a critical mechanism of tumorigenesis. For example, TP53INP1 is significantly down-regulated in liver cancer and promotes metastasis [3]. Protein Phosphatase 2A (PP2A) works with protein kinase to maintain the dynamic balance between states of protein phosphorylation and dephosphorylation. Each PP2A consists of one regulatory B subunit, one structural subunit (PP2A-A) and one core catalytic subunit (PP2A-C) [4]. Of these, PP2A-A and PP2A-C are considered to be core enzymes and exist as dimeric complexes, while B regulatory subunits exist independently. The PP2A regulatory B subunits can be classified into PP2A B55/PR55, B56/PR56/PR61, PR48/PR72/PR130 and PR93/PR110. Isomer molecular weights of these subfamily members are indicated by the designated number with these subfamily numbers indicating the molecular weight of the isomer [5].

Recent studies provide evidence that PR55α plays important roles in oncogenesis and progression of numerous malignancies. PP2A/PR55α (PPP2R2A) regulates several crucial pathways that control cell proliferation and metastasis, including the mitogen activated protein kinase (MAPK) and phosphatidylinositol-3-kinase (PI3K)/AKT pathways as well as the c-Myc, YAP, and apoptosis machinery [6, 7]. PP2A may function as either a tumor suppressor [8, 9] or oncogene [10, 11]. PR55α has been demonstrated to preferentially dephosphorylate phospho-Thr-308 instead of phospho-Ser-473 of the AKT signal pathway in the regulation of cell proliferation and survival of lymphoid cells [12]. In pancreatic ductal carcinoma, elevated PR55α induces cancer cell proliferation through activation of many oncogenic signaling pathways, including ERK, AKT, and Wnt [13]. In lung non-small cell carcinoma, PR55α was frequently found at suppressed levels and directly dephosphorylated ATM at S1981, S189 and S367 to promote its presence at double-strand break sites [14].

Our previous study showed that long noncoding RNA GMAN promoted the phosphorylation of eukaryotic translation initiation factor 4B (eIF4B) at serine-422 by preventing the combination of PPP2R2A (PR55α) and eIF4B [15]. EIF4B is a key component of translation initiation and its activity is controlled by MAPK and PI3K pathways [16]. PR55α dephosphorylated eIF4B-Ser422 and repressed mRNA translation and anti-apoptotic protein expression. PR55α is also reported to negatively regulate the AKT pathway in HCC [17].

This investigation aims to characterize the role of PR55α as a tumor suppressor in HCC. Its expression appeared to possess a strong correlation with HCC recurrence and poorer prognosis. Physiologically, PR55α inhibited cell proliferation and metastasis by repressing the MAPK/AKT pathway.

## Methods

### Clinical tissues and tissue microarrays

HCC tissues and matched normal samples from The Affiliated Huai’an NO.1 People’s Hospital of Nanjing Medical University were used for western blot. Immunohistochemistry (IHC) analysis was performed on tissue microarray kits comprising of 80 human HCC tissues and 80 adjacent non-tumor tissues that were purchased from Shanghai Outdo Biotech Co. Ltd (HLivH160CS01) (National Human Genetic Resources Sharing Service Platform, Shanghai, China). The diagnosis of HCC was confirmed by pathology. Written informed consent was signed by patients and all experimental protocols were reviewed by the Medical Ethics Committee of Shanghai, the People’s Republic of China.

### Cell culture and RNA interference by shRNA

Five HCC cell lines and one normal liver cell line L02 were purchased from KeyGen (Nanjing KeyGen Biotech Co, Ltd, Jiangsu, China). Cells were maintained in 10 % fetal bovine serum(FBS)-supplemented Dulbecco’s Minimum Essential Medium (DMEM) along with antibiotics in an atmospheric condition of 5 % CO_2_ and at 37 °C. Three siRNAs were purchased from Gene Pharma (China). PR55α target sequences utilized in this experiment were the following: GCCUAUGGAUCUAAUGGUUTT for siPR55α#1, GCAGAUGAUUUGCGGAUUATT for siPR55α#2, and GGAAACAUACCAGGUGCAUTT for siPR55α#3. Transfection was performed as previously documented [15].

### Western blotting

Western blotting assays were conducted as previously reported [18]. The primary antibodies were as follows PR55α (1/1000, A2185, Abclonal, China), AKT (1/1000, A17909, Abclonal, China), p-AKT-T308 (1/1000, AP0304, Abclonal, China), P-AKT-S473 (1/1000, AP0140, Abclonal, China), ERK1/2 (1/1000, A4782, Abclonal, China) and p-ERK1/2-T202/Y204 (1/1000, AP0472, Abclonal, China).

### Immunohistochemistry (IHC)

The slices were deparaffinized in xylol, heated for antigen retrieval with sodium citrate (pH 6.0), and treated by hydrogen peroxide. Subsequently, tissue microarray were incubated overnight with anti-PR55α at 4 ℃. Finally, secondary antibody and DAB chromogentic agent were added. PR55α staining intensity was quantified based on the following scale: 0 (staining not detectable), 1 (faintly yellow, weak staining), 2 (light brown moderately staining), and 3 (brown, strongly staining). A high PR55α expression was marked by colour grades of ≥ 2. The staining results were assessed by independent senior pathologists who remained isolated from each other throughout the experiment.

### Cell counting and colony formation assay

Colony formation assays and Cell Counting Kit-8 (CCK-8) assays were used to assess the ability of cell proliferation. 96-well plates were used to house transfected HCC cells at a density of 1 × 10^3^ cells per well. After culturing the cells overnight, cells then received a dose of CCK-8 reagent (Dojindo, Shanghai, China). All treatments were administered daily at the same time. Two hours later, a microplate reader was used to assess the absorbance values (OD).

Six-well plates were used to house the transfected cells (1 × 10^3^ cells per well) which were then allowed to undergo a 10-day incubation period. Colonies that contained ≥ 50 cells were selected for counting. Phosphate buffered saline (PBS) was used to rinse the colonies before they were fixed for 30 min with 4% paraformaldehyde and exposed for 2 min to crystal violet. Three individual repeats of each experiment were done.

### Cell migration and invasion assay

Cell invasion and migration were measured by transwell assays. 200 μl serum-free medium was used to resuspend 2 × 10^4^ transfected HCC cells before they were placed in the upper cartridge (Milllicell, USA) which was precoated with or without 50 μl matrigel. The lower cartridge contained 600 ml DMEM medium with 20% FBS. The cells were then allowed to incubate for 24 h. Paraformaldehyde was then used to fix cells before they were staining using crystal violet. Cells were then photographed and quantified using a microscope for data analysis. The results represented the mean values of three independent experiments.

### Cell cycle and apoptosis assay

Cell cycle and apoptosis assays were conducted using flow cytometer (Beckman Coulter). For cell cycle analysis, transfected cells were harvested and fixed overnight in 70 % ethanol at 4 °C. The cells were then exposed to RNase A before being labeled for 30 min with propidium iodide (PI) at 37 °C.

Cells were collected and stained with Annexin V-FITC and PI reagents prior to the cell apoptosis analysis. Three separate repetitions were carried out for each experiments.

### In vivo tumor assay

BABL/c nude mice were obtained from Nanjing Medical University (Nanjing, Jiangsu, China). Six-week-old female nude mice (n = 5) were treated over their flanks with subcutaneous injections of approximately 2 × 10^6^ LM3 cells which either possessed stable PR55α knockdown or PR55α control. The xenograft tumors were dissected and imaged at 4 weeks post-inoculation. Terminal deoxynucleotidyl transferase (TdT) mediated dUTP nick end labelling (TUNEL) staining and Ki-67 immunofluorescence staining were performed as described previously [15]. For experiments regarding lung metastases, the same cancer cells were administered intravenously into the tail veins of nude mice (n = 5). Lung nodules were measured under a dissecting microscope at 40 days after treatment. The Experimental Animal Management Committee of Nanjing Medical University approved all mice experiments which were formulated in strict compliance to established guidelines.

### RNA sequencing

RNA sequencing analysis and quantification were utilized to assess changes in mRNA profiles of LM3 cells with PR55α knockdown or PR55α control. Samples were performed in triplicate. The differentially expressed mRNAs were selected with fold change < 0.5 or > 2 and possessed a P value < 0.05 by R package edgeR. We also analyzed GO enrichment and KEGG enrichment in the differentially expressed mRNAs.

### Statistical analysis

Mean ± standard deviation was used to express all collected data. Analysis was carried out using GraphPadPrism 6. Variances between normal and HCC samples were contrasted using paired t-test. The associations between clinicopathological features and PR55α expression were assessed by the fishers exact test and Chi-square test. Statistical significance was designated as follows: *P < 0.05, **P < 0.01, ***P < 0.001 and ****P < 0.001. P < 0.05 was interpreted as a result that was statistically significant.

## Results

### PR55α is downregulated in HCC tissues in contrast to healthy specimens

**Fig. 1:**
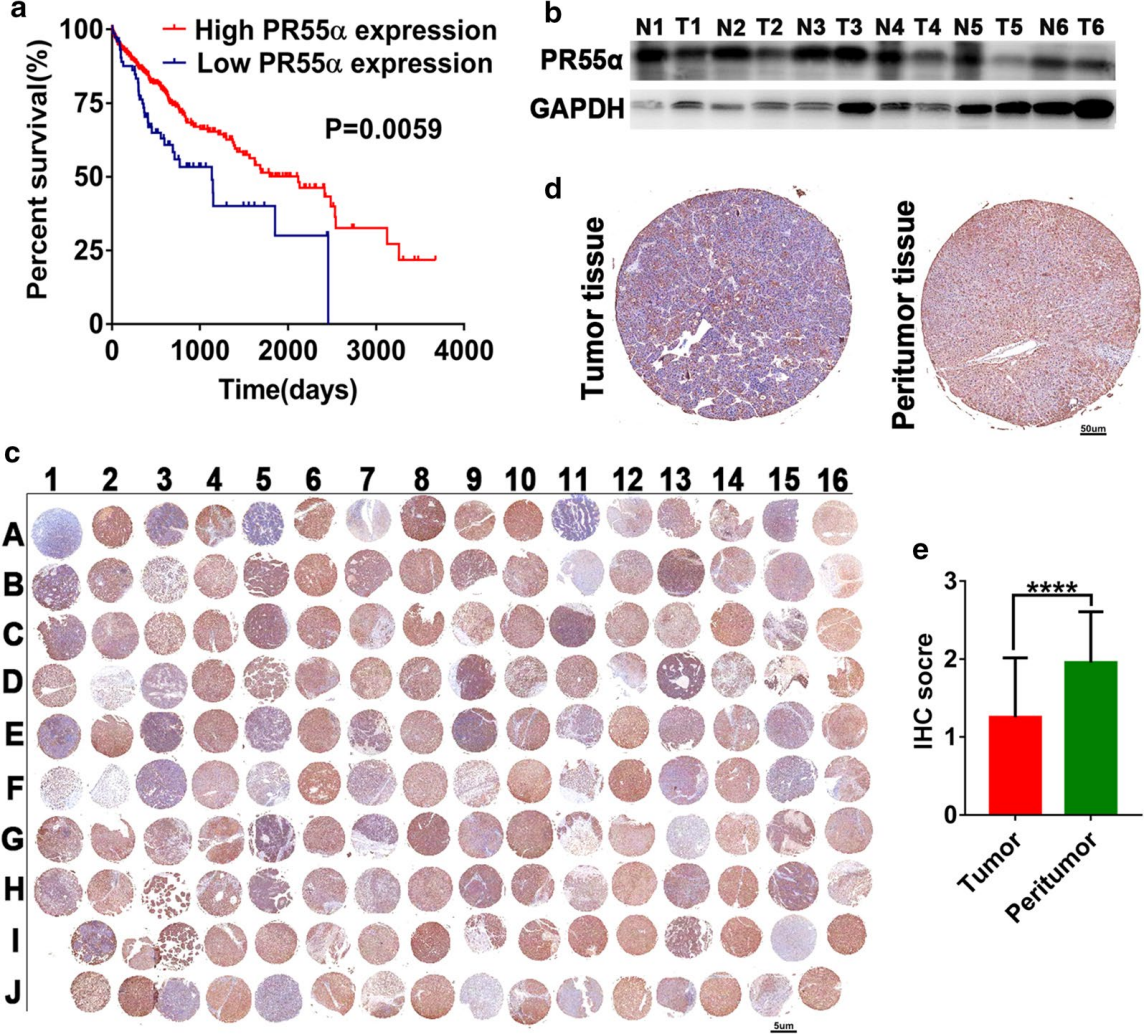
HCC tissues that expressed lower PR55α levels were noted in patients with poorer prognoses. **a** The overall survival of patients with either high (n = 82) or low (n = 283) PR55α expressions (Data extracted from The Human Protein Atlas) (P = 0.0059). **b** PR55α expressions in six paired of HCC tissues and non-adjacent normal liver tissues as determined using Western blotting. **c** Images of immunohistochemical (IHC) staining of PR55α in 80 paired HCC tumor tissue and peritumor tissue. (3× magnification). The odd rows represent HCC tissues and the even rows represent peritumor tissues. **d** Representative IHC-stained images of PR55α in HCC tumor tissue and healthy liver tissues samples. (40× magnification). **e** IHC scores of PR55α in HCC and matched healthy liver samples (P < 0.0001)

To investigate the potential function of PR55α,we assessed the relationship between PR55α and HCC prognosis. As shown in Fig. 1a, lower PR55α expression levels were strongly linked to an overall poorer survival (P = 0.0059). Likewise, western blotting assays also demonstrated a lower expression of PR55α in HCC samples (Fig. 1b). We next evaluated PR55α expression by IHC in a tissue microarray comprising of 80 HCC tissues and adjacent healthy tissues. Our tissue microarray results showed that 57.5 % (46/80) HCC tissues demonstrated weak or no PR55α expression compared with 36.25 % (29/80) cases in healthy tissues surrounding the tumor (Fig. 1c, d). IHC analysis found that PR55α expression in HCC tissues were significantly lower in comparison to healthy samples (P < 0.0001) (Fig. 1e) (Additional file 1: Table S1).

In order to explore the potential clinical value of PR55α expression, the clinical characteristics of 80 HCC patients were analyzed. Correlation analysis of clinical parameters showed that PR55α expression correlated inversely with TNM stage and vascular invasion (Table 1).

**Table 1:** The association between PR55α expression and clinical pathological data in HCC patients

Features	PR55α level		P
	High expression	Low expression	
Cases	34	46	
Age (years)			
< 60	18	30	0.268
≥ 60	16	16	
Gender			
Man	30	38	0.544
Female	4	8	
Cirrhosis			
Yes	25	36	0.791
No	9	10	
Size (cm)			
< 5	5	5	0.736
≥ 5	29	41	
TNM			
I–II	18	12	0.020*
III	16	34	
Microvascular invasion			
Yes	5	24	0.010*
No	29	22	
Histologic grade			
Low	3	4	0.890
Middle	17	22	
High	14	20	

*P < 0 0.05

### PR55α knockdown induces HCC cell growth and metastasis

**Fig. 2:**
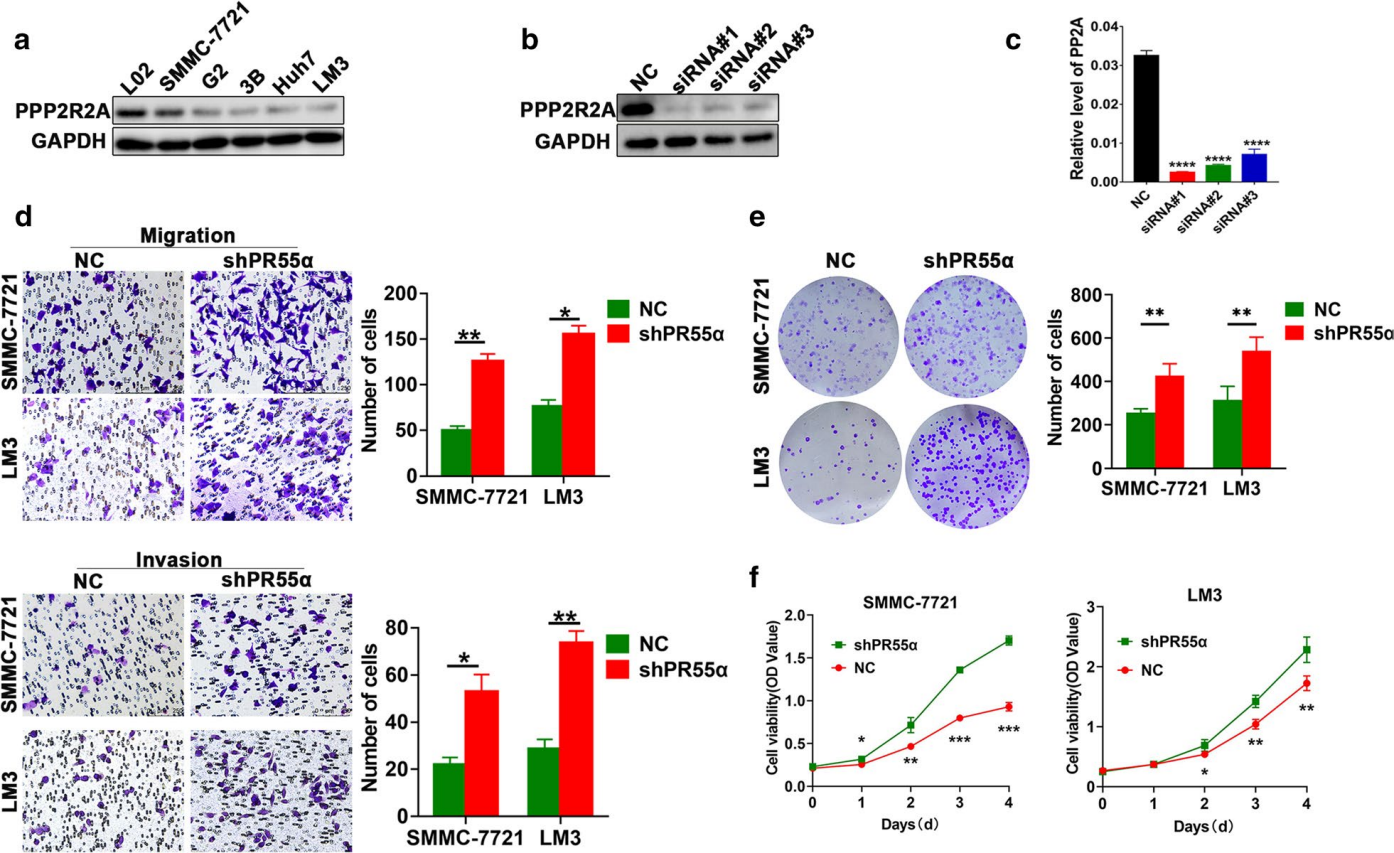
Knockdown of PR55α expression induces cell proliferation and increases cell migration and invasion. **a** PR55α expressions across one normal liver cell line and five lines of liver cancer cells as demonstrated using western blot. siRNA efficacy in knocking down PR55α as tested by western blot (**b**) and real-time PCR (**c**). **d** Migration (above) and invasion (down) assays in SMMC-7721 and LM3 lines (200× magnification). **e** Colony formation assays for liver cancer cells transfected with shPR55α. **f** Growth curves in PR55α control and knockdown cells. Each experiment was an average of results obtained from three replicates and is depicted in terms of mean ± SD. *P < 0.05, **P < 0.01, ***P < 0.001

In order to fully illustrate the role of PR55α in HCC cell function, Western blotting experiments were done to quantify HCC cell PR55α expression levels. As shown in Fig. 2a, PR55α exhibited a higher expression level in SMMC-7721 and LM3 HCC cell lines as compared to L02 normal liver cells. We then used specific siRNA targeted against PR55α in LM3 cells and verified the transfection efficiency using immunoblotting assays and real-time PCR (Fig. 2b, c). Stably transfected HCC cell lines were then used for subsequent experiments. Knockdown of PR55α boosted the migration and invasion ability of both SMMC-772 and LM3 compared with control cells, as evidenced by transwell assays (Fig. 2d). Moreover, colony formation assays and CCK8 assays indicated that PR55α-shRNA significantly promoted proliferation of SMMC-772 and LM3 cells in comparison to control cells (Fig. 2e, f). When interpreted as a whole, we conclude that the migratory and proliferative ability of HCC cells were attenuated by PR55α.

### PR55α knockdown suppresses apoptosis and cell cycle arrest in HCC cells

**Fig. 3:**
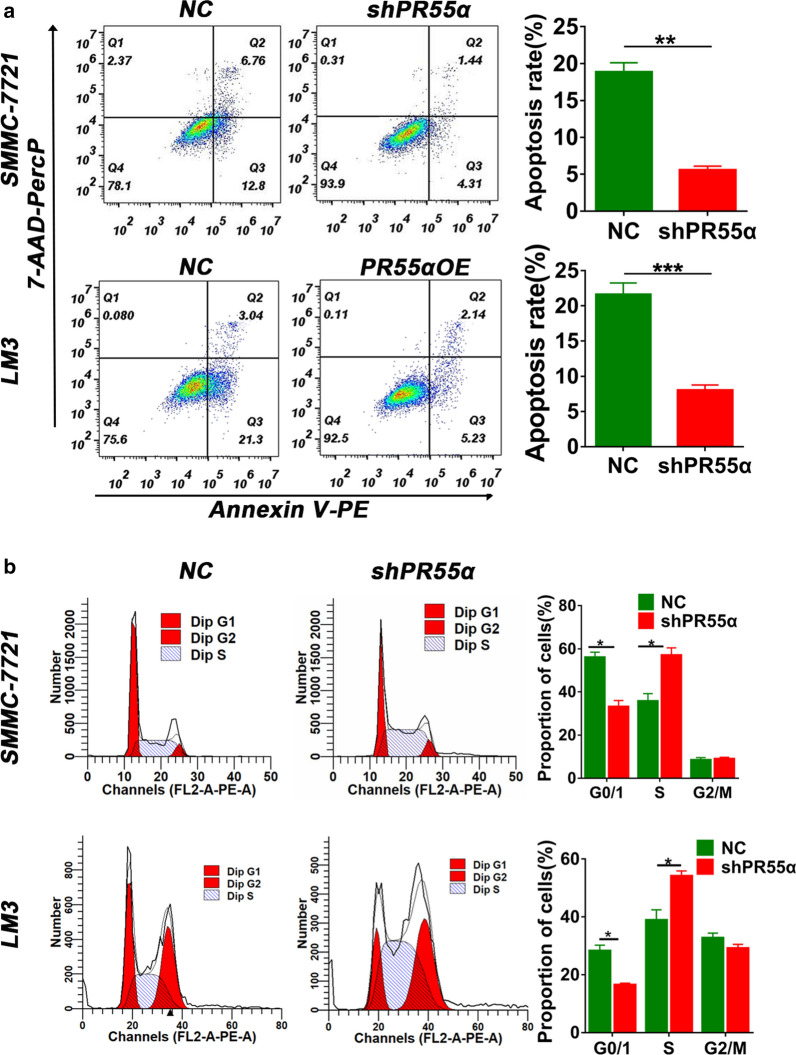
PR55α silencing represses cell cycle arrest and apoptosis. **a** Downregulation of PR55α expression inhibits apoptosis in SMMC-7721 and LM3 cells in contrast to the NC group (P < 0.05). **b** The cell cycles of LM3 and SMMC-7721 cells upon transfection with shPR55α and NC were analyzed using a flow cytometer (P < 0.05)

To further assess the inhibitive function of PR55α,a fluorescence-activated cell sorting (FACS) analysis was done to determine the proportion of SMMC-7721 and LM3 cells in each phase of the cell cycle as well as the percentage that were apoptosed. We discovered that PR55α knockdown significantly decreased the amount of cells undergoing apoptosis (Fig. 3a) (P = 0.009 for SMMC7721 and P = 0.0002 for LM3). As illustrated in Fig. 3b, PR55α-shRNA induced significantly decreased cells in the G0/1 phase while increasing the number of cells in the S phase (P < 0.05).

### Decrease of PR55α promotes cell growth and metastasis in vivo

**Fig. 4:**
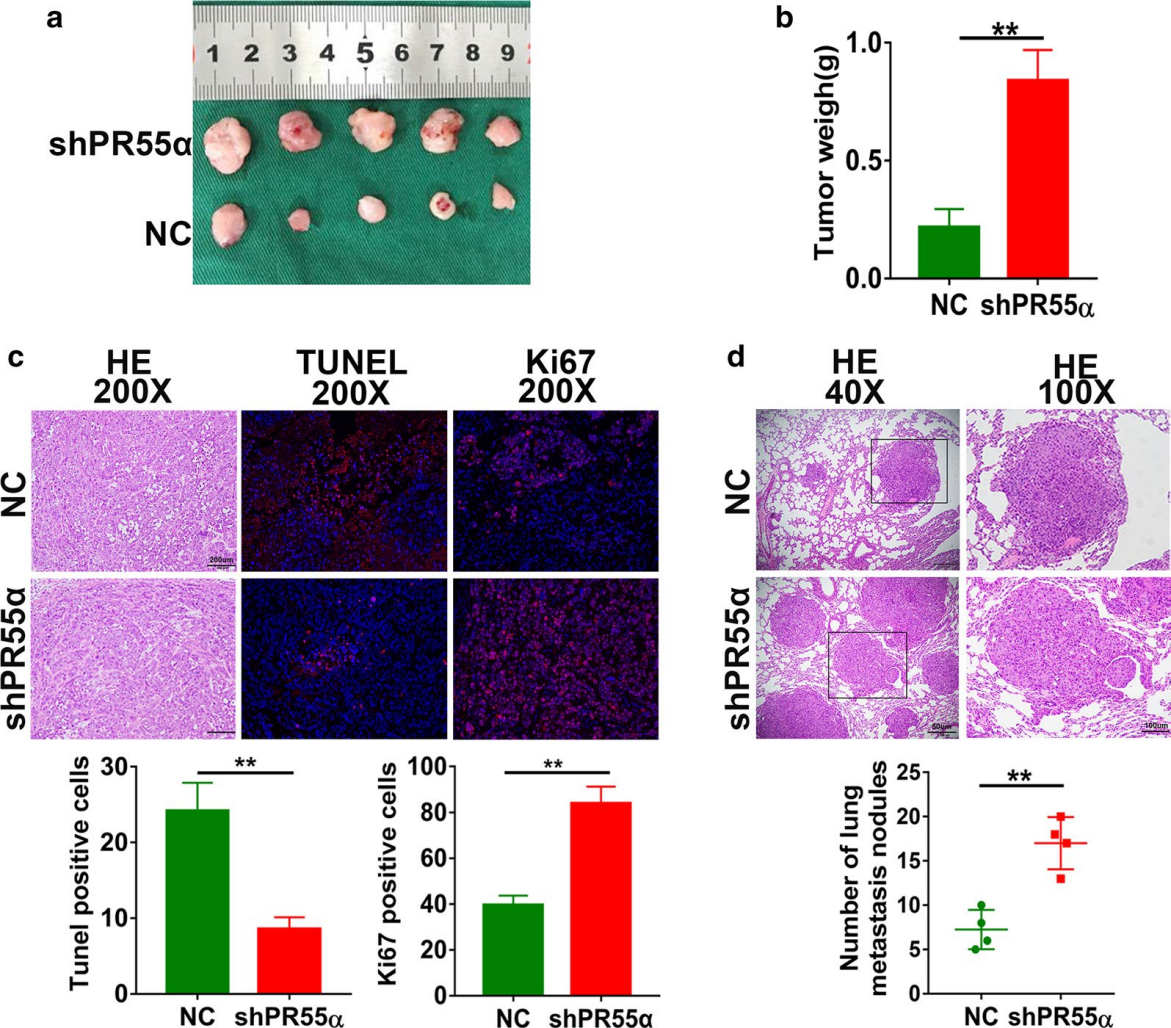
Decrease of PR55α promotes in vivo cell metastasis and growth. **a** Xenograft tumor images in nude mice after 4 weeks of growth (n = 5). **b** Evaluation of tumor weights in PR55α control and knockdown groups. **c** Representative images of the tumors grown on nude mice with HE, TUNEL and Ki-67 stainings (above). Representative images of HE, Ki-67 and TUNEL staining in tumor xenografts (left). Red signal stains was ki67 positive cells and apoptotic cells. Evaluation of TUNEL positive areas (down right) (P = 0.002) and ki67 expression level (down left) (P = 0.001) was assessed. **d** Lung metastasis was embedded in paraffin and stained with HE (above). The number of lung metastasis nodules was measured (down) (P = 0.004)

We next evaluated the inhibitive function of PR55α on liver cancer growth and metastasis in vivo. Male nude mice were subjected to subcutaneous injections of LM3 cells that stably expressed either PR55α-shRNA or PR55α-control. Our results showed that PR55α-shRNA cells developed into larger tumors than control cells in nude mice (Fig. 4a). The weights of tumor xenografts also indicated that PR55α knockdown promoted tumor growth compared with the control cells (Fig. 4b) (P = 0.005). Xenograft tissues were also subjected to H&E/ki67 staining (P = 0.001) and TUNEL assays (P = 0.002) (Fig. 4c). As shown in Fig. 4c, shPR55α tumor cells demonstrated a significantly decreased proportion of apoptotic DNA fragments and elevated Ki-67 index compared with the control group. In addition, more pulmonary metastases were found in the shPR55α group (Fig. 4d) (P = 0.004). Based on this information, we conclude that the severity of HCC malignancy is inhibited by PR55α both in vitro and in vivo.

### PR55α inhibits HCC progression through MAPK and AKT signaling

**Fig. 5:**
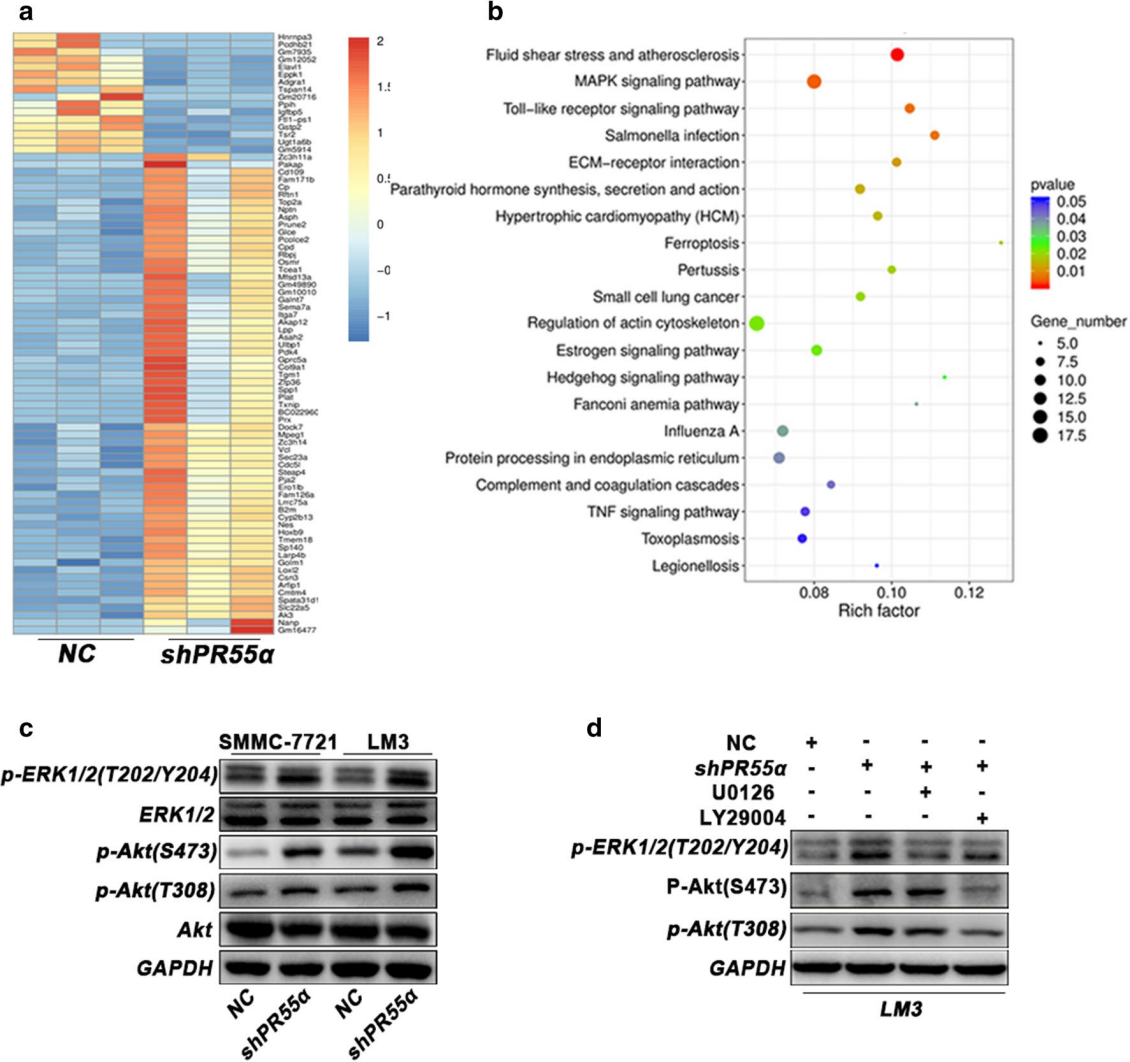
Knockdown of PR55α by shRNA activates AKT and ERK1/2 signaling. **a** Microarray analysis was conducted to screen the mRNAs which were subjected to PR55α regulation. **b** KEGG pathway analysis revealed the potential signaling pathways involved in PR55α. **c** AKT/ERK signaling pathway protein expressions in HCC cells transfected with shPR55α or shControl were evaluated using western blot. **d** The indicated proteins of AKT/ERK signaling pathway were detected with or without AKT inhibitor LY294002 (20 μM) and MEK inhibitor U0126 (15 μM)

To explore the molecular mechanisms of PR55α in HCC, a microarray analysis was performed in LM3 cells with PR55α knockdown and in control cells. The results revealed 166 down-regulated mRNAs and 754 up-regulated mRNAs (Additional file 2: Table S2). We also uncovered various differentially expressed genes (Fig. 5a). Kyoto Encyclopedia of Genes and Genomes (KEGG) pathway analysis of the genes that were found to be upregulated in PR55α silenced cells suggested a potential association between PR55α and MAPK signaling pathways (Fig. 5b). Previous investigations have noted that PR55α was significantly involved in the AKT signaling pathway [17]. Sh-PR55α notably increased the amount of phosphorylated AKT-S473, AKT-T308 as well as ERK1/2-T202/Y204 in SMMC-7721 and LM3 cells, as evidenced by Western blotting (Fig. 5c). To verify this phenotype, we treated above cells with the PI3K inhibitor LY294002 and the MEK inhibitor U0126. The results showed that LY294002 and U0126 can reverse PR55α-induced alteration of phosphorylated ERK1/2 or AKT (Fig. 5d). Together, these results suggested a vital role for PR55α in aberrant MAPK and AKT signaling pathway activation in HCC.

## Discussion

PP2A is a serine/threonine phosphatase which possesses a rather controversial role in tumorigenesis. PR55α (encoded by PPP2R2A) can function as both an oncogene and tumor suppressor in different cancers. Previous studies revealed that knockdown of PR55α hindered non-small cell lung cancer cellular growth by increasing JUN T239 phosphorylation [18]. However, more studies supported the tumor suppressing role of PR55α. Cancer cell invasion and proliferation have been noted to be suppressed by PR55α in diffuse large B-cell lymphoma cells [19] and thyroid cancer [20]. Divergences of transcriptional and posttranscriptional regulation of PR55α were the possible reasons for this discrepancy. High-resolution analyses of somatic copy number confirmed that significant loss-of heterozygosity (LOH) of PPP2R2A-containing region lead to down-regulation of PPPP2R2A mRNA expression in many human cancers [21]. The percent of samples with decreased PPP2R2A mRNA expression in lung cancer, thyroid gland cancer, breast cancer and HCC were 36.82%, 94.44%, 65.21% and 58.82% respectively. However, only 20% of PPP2R2A mRNA expression was reduced in pancreatic cancer. Additionally, the expression of PPP2R2A is also regulated by multiple MirRNAs. For example, MiR-222 can suppress PPP2R2A expression and promote the proliferation or invasion of papillary thyroid cancer [20], diffuse large B-cell lymphoma [19] and HCC [17]. But, miR-665 was reported to promote PPP2R2A overexpression and inhibit the proliferation and epithelial–mesenchymal transition of gastric cancer cells [22]. Specifically, PR55α was lowly expressed in lung cancer, thyroid gland cancer, breast cancer, diffuse large B-cell lymphoma and HCC, but highly expressed in pancreatic cancer and gastric cancer. To our knowledge, this is the first study indicating PR55α expressions were frequently at low expression and acted as a regulator of cell proliferation and metastasis in HCC.

In current research, PR55α expression was found to be suppressed in HCC tissues, and the negative correlation between PR55α down regulation, several malignant characteristics and poor prognosis was confirmed. Functionally, PR55α knockdown significantly triggered cell proliferation and invasion, induced cessation of cell cycle progression and facilitated both in vivo and in vitro cell apoptosis. Furthermore, using both microarray analysis and western blot, we demonstrated that sh-PR55α could activate the MAPK/AKT signaling pathway. All these results indicate that PR55α is a tumor suppressor in HCC and may be a reliable biomarker as well as a means to facilitate earlier HCC diagnosis and more effective treatment.

Phosphatases provide both positive and negative regulation for the MAPK pathway at various points. Previous studies showed that PP2A/PR55α facilitated ERK1/2 phosphorylation/activation by activating KSR and Raf [10, 23]. However, PR55α has been reported to negatively regulate RAS signaling [24]. PR55α has been reported to inhibit phosphorylation of ERK1/2 in non-small cell lung cancer cells [14] and vascular smooth muscle cells [25]. Our studies showed that knockdown of PR55α significantly induced MAPK signaling. Consistent with the microarray analysis, western blot showed that shPR55α promoted Erk1/2 phosphorylation.

In HCC, PI3K/AKT pathway activation represents a significant oncogenic process that has been documented to be commonly activated in HCC [26]. Complete activation of AKT required phosphorylation at Ser473 by mTORC2 and at Thr308 by PDK1 [27, 28]. AKT is activated by various kinases, such as PKA, ACK1 and TNK2, and is inhibited by a variety of phosphatases, such as PP2A, PTEN, PHLPPs and INPP4B [6, 29]. A previous study confirmed that PR55α can directly bind and induce preferential dephosphorylation of phospho-Thr-308 instead of phospho-Ser-473, as evidenced by in vitro assays on dephosphorylation using both NIH3T3 and FL5.12 cells [30]. However, PR55α silencing increased baseline phosphorylation of AKT-Thr308 and inhibited Akt-Ser473 phosphorylation upon exposure to insulin-like growth factor-1 in H9c2 cells [31]. More importantly, PR55α significantly regulated AKT phosphorylation at the Thr-308 and Ser-473 residues in pancreatic cancer cells [13] and liver cancer cells [17]. The discrepancy among these previous findings may be due to the differences in cell type or tumor type. Here, our results suggest that decreased PR55α expression is responsible for increased AKT-Ser473 and AKT-Thr308 phosphorylation in HCC.

Some limitations need to be pointed out here. First, we failed to analyze the effect of PR55α on OS and PFS after surgery due to the lack of follow-up data on tissue microarray. Second, PP2A comprises of PP2A-A, PP2A-B and PP2A-C. But, we did not detect the expression of other subunits of PP2A in HCC in this article. Lastly, previous studies showed PR55α can regulate multiple oncogenic signaling pathways via dephosphorylation of key gene sites. In this article, only transcriptome sequencing may not fully reveal the potential carcinogenic effects of PR55α in HCC. All these questions need further study to investigate. In the next step, phosphorylation analysis with or without PR55α knockdown may provide a better understanding of its underlying mechanisms of action and phosphorylation sites.

## Conclusion

In conclusion, our results highlight a potential prognostic value of PR55α, and PR55α inhibits proliferation and metastasis of HCC cells likely by inactivating MAPK/AKT signaling.

## Supplementary Information

**Additional file 1: Table S1.** IHC score of PR55α expression in HCC tissues and paired healthy samples.

**Additional file 2: Table S2.** The results of microarray analysis showed 166 down-regulated mRNAs and 754 up-regulated mRNAs.

## Data Availability

Related data and materials could be seen in the manuscript and the additional files.
